# Determining clinically meaningful decline in preclinical Alzheimer disease

**DOI:** 10.1212/WNL.0000000000007831

**Published:** 2019-07-23

**Authors:** Philip S. Insel, Michael Weiner, R. Scott Mackin, Elizabeth Mormino, Yen Ying Lim, Erik Stomrud, Sebastian Palmqvist, Colin L. Masters, Paul T. Maruff, Oskar Hansson, Niklas Mattsson

**Affiliations:** From the Center for Imaging of Neurodegenerative Diseases (M.W., R.S.M.), Department of Veterans Affairs Medical Center; Departments of Radiology and Biomedical Imaging (P.S.I., M.W.) and Psychiatry (P.S.I., R.S.M.), University of California, San Francisco; Clinical Memory Research Unit, Faculty of Medicine (P.S.I., E.S., S.P., O.H., N.M.), Memory Clinic (E.S., S.P., O.H.) and Department of Neurology (N.M.), Skåne University Hospital, and Wallenberg Center for Molecular Medicine (N.M.), Lund University, Sweden; Department of Neurology and Neurological Sciences (E.M.), Stanford University, CA; The Florey Institute (Y.Y.L., C.L.M., P.T.M.), The University of Melbourne; and CogState (P.T.M.), Melbourne, Australia.

## Abstract

**Objective:**

To determine the time required for a preclinical Alzheimer disease population to decline in a meaningful way, use estimates of decline to update previous clinical trial design assumptions, and identify factors that modify β-amyloid (Aβ)–related decline.

**Methods:**

In 1,120 cognitively unimpaired individuals from 3 international cohorts, we estimated the relationship between Aβ status and longitudinal changes across multiple cognitive domains and assessed interactions between Aβ and baseline factors. Power analyses were performed to explore sample size as a function of treatment effect.

**Results:**

Cognitively unimpaired Aβ+ participants approach mild cognitive impairment (MCI) levels of performance 6 years after baseline, on average. Achieving 80% power in a simulated 4-year treatment trial, assuming a 25% treatment effect, required 2,000 participants/group. Multiple factors interacted with Aβ to predict cognitive decline; however, these findings were all cohort-specific. Despite design differences across the cohorts, with large sample sizes and sufficient follow-up time, the Aβ+ groups declined consistently on cognitive composite measures.

**Conclusions:**

A preclinical AD population declines to the cognitive performance of an early MCI population in 6 years. Slowing this rate of decline by 40%–50% delays clinically relevant impairment by 3 years—a potentially meaningful treatment effect. However, assuming a 40%–50% drug effect highlights the difficulties in preclinical AD trial design, as a more commonly assumed treatment effect of 25% results in a required sample size of 2,000/group. Designers of preclinical AD treatment trials need to prepare for larger and longer trials than are currently being considered. Interactions with Aβ status were inconsistent and not readily generalizable.

To effectively alter the course of Alzheimer disease (AD), interventions may need to occur during the preclinical stage of the disease, before the onset of clinical symptoms.^1^ Demonstrating that treatments are effective during the preclinical stage will require understanding the magnitude of early β-amyloid (Aβ)–related cognitive decline in cognitively unimpaired adults.^2^ Defining meaningful decline will help determine the time frame for subtle cognitive changes to progress to incipient functional decline and to identify an optimal treatment window.

The association between Aβ status and cognition in preclinical AD varies widely.^3–9^ The design of the A4 study,^10^ the first clinical trial in preclinical AD, was based on early estimates of Aβ-related decline using the Alzheimer's Disease Neuroimaging Initiative (ADNI)^11^ and the Australian Imaging, Biomarkers & Lifestyle (AIBL) Study.^12^ The effect of Aβ on cognitive decline in AIBL was 4-fold the magnitude of the effect in ADNI, highlighting an inconsistent picture of early cognitive decline and uncertain implications for powering a trial in early AD. Understanding how sampling variation and study design features influence estimates of cognitive decline will optimize the design of trials in preclinical AD.

The aims of this study were to harmonize several large studies in order to (1) determine the time required for a preclinical AD population to decline in a clinically meaningful way, (2) characterize how decline differs by cognitive domain, (3) update previous study design assumptions regarding sample size, power, and the required treatment effect, and (4) identify factors that modify Aβ-related decline.

## Methods

### Standard protocol approvals, registrations, and patient consents

This study was approved by the institutional review boards of all of the participating institutions. Informed written consent was obtained from all participants at each site.

### Participants

Participants from each of the cohorts ADNI, AIBL, and the Swedish Biomarkers for Identifying Neurodegenerative Disorders Early and Reliably (BioFINDER) study^13^ were included if they were classified as cognitively normal at baseline, were tested for Aβ biomarkers (using either CSF or PET), and were followed longitudinally with neuropsychological examinations.^11–13^ Participants were excluded from any of the 3 studies if they had a major neurologic or psychiatric illness or a history of substance abuse. In addition, ADNI participants were excluded if the screening MRI showed evidence of infection, infarction, or other focal lesions, including multiple lacunes or lacunes in a critical memory structure. MRI results were not part of the exclusionary criteria for AIBL or BioFINDER, but BioFINDER participants were excluded if they refused MRI or lumbar puncture. Detailed exclusionary criteria for ADNI can be found at adni.loni.usc.edu/wp-content/uploads/2008/07/adni2-procedures-manual.pdf and for BioFINDER at biofinder.se/biofinder_cohorts/cognitively-healthy-elderly/. We also included 305 participants enrolled into the early mild cognitive impairment (MCI) cohort in ADNI (defined by a subjective memory complaint and a delayed logical memory score of 9–11 for those with 16 or more years of education, 5–9 for 8–15 years of education, or 3–6 for 0–7 years of education, where possible scores range from 0 to 25)^14^ for a comparative analysis. The extensions of ADNI introduced the distinction of MCI into early and late MCI in the attempt to define an earlier point in time for disease detection. Late MCI refers to the original definition of MCI (performance for 1.5 SD below the normative mean), whereas in early MCI, impairment is defined as performance between 1.0 SD and 1.5 SD below the normative mean on a standard test. Because of recent evidence of an artificially low reversion rate from MCI to control in ADNI,^15^ we excluded 7 early MCI participants who consistently had a global Clinical Dementia Rating (CDR) score of zero after screening in a sensitivity analysis.

Data on memory complaints in the controls were available in AIBL and ADNI. In AIBL, participants with a memory complaint were identified by the response to the question, “Do you have difficulties with your memory?” In ADNI, the participant was required to have a significant memory concern as reported by the participant, study partner, or clinician and a score >16 on the first 12 items of the Cognitive Change Index.

### Aβ biomarkers

Aβ status was defined by PET imaging if available (all AIBL and a majority of ADNI participants), and otherwise by CSF biomarkers (all BioFINDER and a small proportion of ADNI participants). PET imaging was done using ^18^F-florbetapir PET in ADNI and using ^18^F-florbetapir, ^11^C–Pittsburgh compound B (PiB), or ^18^F-flutemetamol PET in AIBL. Methods to acquire and process imaging data were described previously.^16–18^ CSF samples were collected at baseline by lumbar puncture. CSF methods have been described previously.^19–21^ In short, ADNI CSF samples were analyzed for CSF Aβ42 using the AlzBio3 assay (Fujirebio, Ghent, Belgium) on the xMAP Luminex platform. BioFINDER CSF samples were analyzed for CSF Aβ42 and Aβ40 using ELISA assays (ADx/EUROIMMUN AG, Lübeck, Germany). For ADNI participants, Aβ+ was defined as ^18^F-florbetapir PET standardized uptake*value*ratio (*SUVR*) >1.1 (n = 381)^22^ or CSF Aβ42 <192 ng/L (n = 62).^19^ For AIBL, Aβ+ was defined as ^18^F-florbetapir PET SUVR >1.1 (n = 72), ^11^C-PiB PET SUVR >1.5 (n = 201), or ^18^F-flutemetamol SUVR >0.62 (n = 75).^23^ In BioFINDER, Aβ+ was defined as CSF Aβ42/Aβ40 <0.1.^24^

### Cognitive testing

Participants were followed for up to 6 years for neuropsychological testing. In ADNI, tests were administered annually with an additional test at month 6 for most measures. In AIBL, tests were administered every 18 months. In BioFINDER, tests were administered every 2 years. The Preclinical Alzheimer's Cognitive Composite (PACC)^25^ and its individual components were the primary outcomes compared in the 3 cohorts. This composite was developed specifically to be sensitive to early cognitive changes in AD and is being incorporated in clinical trials of disease-modifying treatments.^10^ Substitutions representing the same cognitive domain were made in the case where the original PACC components were not available or had limited follow-up in a cohort's neuropsychological battery, following previous procedures.^10,25^ Visits where all components or substitutions were available were included. For ADNI, the modified PACC comprised the Mini-Mental State Examination (MMSE), Logical Memory Delayed Recall (dMemory), Trail-Making Test B (Trails B), and the Delayed Word Recall from the Alzheimer's Disease Assessment Scale–Cognitive Subscale (dADASc). For AIBL, the PACC was constructed using the MMSE, dMemory, Digit Symbol Substitution Test, and the Delayed Recall from the California Verbal Learning Test (dCVLT). For BioFINDER, the PACC consisted of the MMSE, dADASc, and Trails B. To calculate the composite, *z* scores of the individual components were taken over all time points and then summed. This sum was then standardized to the mean and SD of the baseline score of the sum.

The PACC includes 2 measures of delayed memory recall; however, because only one delayed memory measure was available in BioFINDER, dADASc was given twice the weight in BioFINDER to reflect the contribution of delayed memory recall in the composite. Immediate recall (logical memory for ADNI and AIBL, Alzheimer's Disease Assessment Scale–Cognitive Subscale word recall for BioFINDER) was evaluated as a measure of baseline memory ability to predict changes in the PACC. The CDR sum of boxes (CDR-SB) was also evaluated as an outcome measure.

### Statistical analysis

Longitudinal measures were modeled using generalized least squares regression assuming a compound symmetric covariance structure.^26^ To capture departures from linearity in the trajectory of the neuropsychological measures, continuous time from baseline test was parameterized using restricted cubic splines.^27^ Cubic splines are functions of polynomials allowing flexibility in the estimation of trajectories over time. Time was modeled with 3 spline knots, 2 at the boundaries and 1 at median follow-up. Differences in trajectories between Aβ+ and Aβ− groups were tested using interactions between the 2 measures for time and the group factor using likelihood ratio tests and change in the Akaike information criterion (AIC), a model selection tool.^28^ A lower value of AIC indicates a better fitting model. Baseline age was also modeled using restricted cubic splines to capture its nonlinear effect on cognition. Models included the 2 spline-estimated measures for baseline age; sex; years of education, where education was categorized as 0–12 years, 13–15 years, and 16 or more years; the interaction between Aβ status and the 2 measures for time; and the main effects for Aβ status and time.

We also evaluated interactions between Aβ status and demographics (baseline age, sex, education), *APOE* (presence of at least one ε4 allele), memory complaint, and baseline memory, and their effect on changes in the PACC. These models included all the terms described above as well as the 3-way interaction between time, Aβ status, and the demographic term. The interaction with age was evaluated using the 2 spline-estimated measures.

To estimate power for hypothetical clinical trials, mixed models of repeated measures^29^ were used to estimate the variance components of the change from baseline in the PACC for the Aβ+ subjects in each cohort. To mirror current preclinical trial design,^10^ Aβ+ subjects with very high cognitive scores (dMemory >15 for ADNI [n = 32] and AIBL [n = 12] and dADASc >8 in BioFINDER [n = 29]) were excluded in order to remove “supernormals.” This was done to mitigate the inclusion of participants with little or no sign of near-term decline in order to increase the likelihood of decline in the placebo group and improve power. Model estimates were then used to calculate the power for 4- and 6-year clinical trials, assuming a range of sample sizes and drug effects, a 6-month visit interval, and a 30% dropout rate. Individual cohort estimates of change from baseline and variance were then meta-analyzed to get combined estimates of change over time.^30^

In order to provide a context for meaningful clinical decline in the cognitively normal participants, we compared the baseline PACC scores in the normal participants to the PACC scores in the ADNI early MCI participants (stratified by Aβ status). We then evaluated the mean time for the average preclinical AD participant to reach the mean baseline PACC score in the early MCI groups.

Baseline associations between demographics and Aβ positivity were assessed using the Wilcoxon rank-sum test for continuous variables and a χ^2^ test for categorical variables. Reductions of AIC >2 and *p* values <0.05 were considered significant. All analyses were done in R v3.4.3 (r-project.org). GLS models were fit using the gls function from the nlme package.

### Data availability

Data from the ADNI and AIBL cohorts are publicly available. Data from BioFINDER may be requested.

## Results

### Cohort characteristics

A total of 443 cognitively healthy controls from ADNI, 348 from AIBL, and 329 from BioFINDER were included in the study. Aβ+ groups were older, had a higher frequency of *APOE* ε4 positivity, and performed significantly worse on several cognitive tests at baseline, compared to Aβ− groups, in all cohorts (table 1). The proportion of *APOE* ε4 positivity in the Aβ+ group was similar in BioFINDER (55%) and AIBL (53%) and lower in ADNI (44%). Education and sex were not associated with Aβ positivity in AIBL or BioFINDER; however, Aβ+ ADNI participants were more likely to be female and have less education compared to Aβ− ADNI participants. The majority of ADNI participants had 16 or more years of education, whereas the majority of both AIBL and BioFINDER participants had fewer than 16 years of education. There was no association between subjective memory complaint and Aβ status in either ADNI or AIBL (subjective memory complaint data were not available in BioFINDER).

**Table 1:**
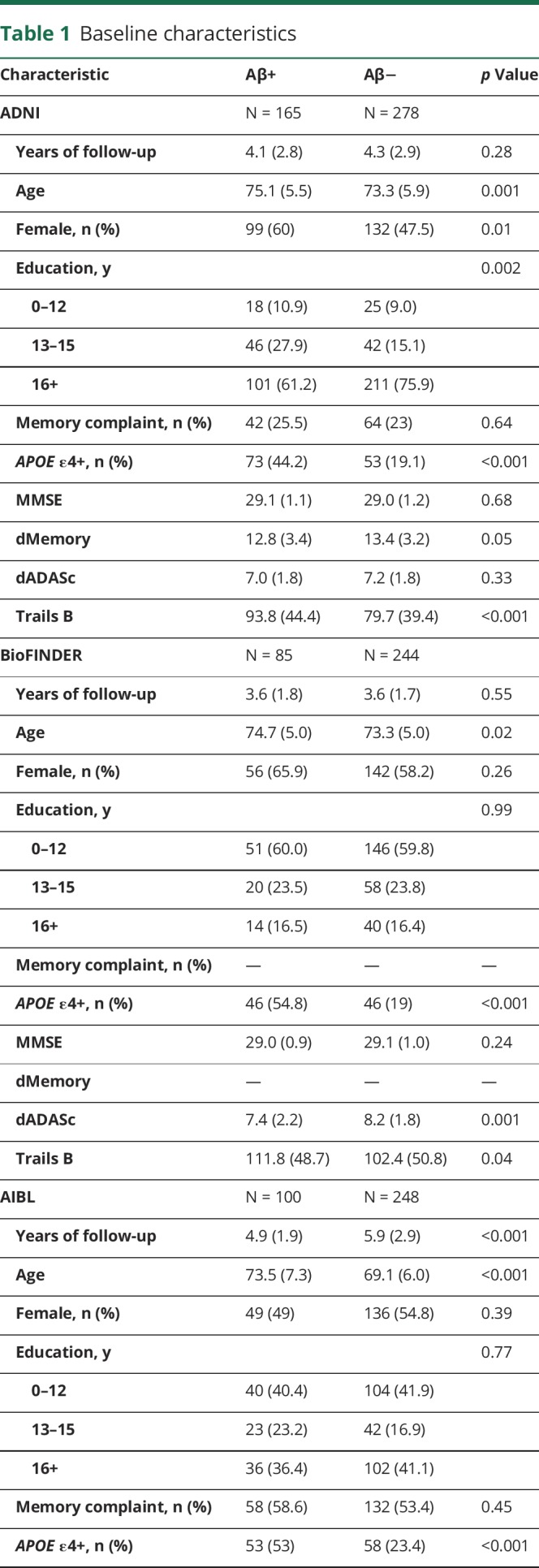

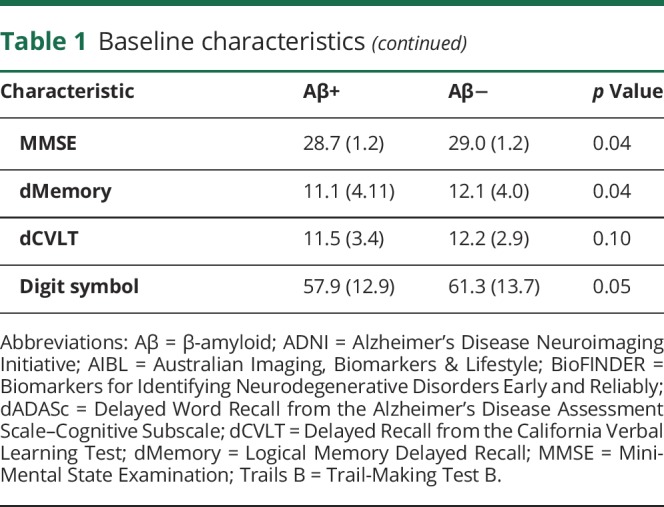
Baseline characteristics

There was considerable variability in attrition rates across the 3 cohorts. At 4 years of follow-up, ADNI retained 46% of its participants; however, dropout was not associated with age, sex, education, Aβ status, or baseline memory performance (*p* > 0.13). At 4 years, BioFINDER retained 69% of its participants. Women were less likely to drop out (odds ratio [OR] = 0.78, *p* = 0.01), participants with more education were more likely to drop out (OR = 1.35, *p* = 0.04), and older age was associated with increased drop out (OR = 1.28 for 1 SD increase in age, *p* < 0.001). AIBL retained 90% of its participants, but older age was associated with increased drop out (OR = 1.26 for 1 SD increase in age, *p* = 0.01).

### Cognitive changes

**Figure 1:**
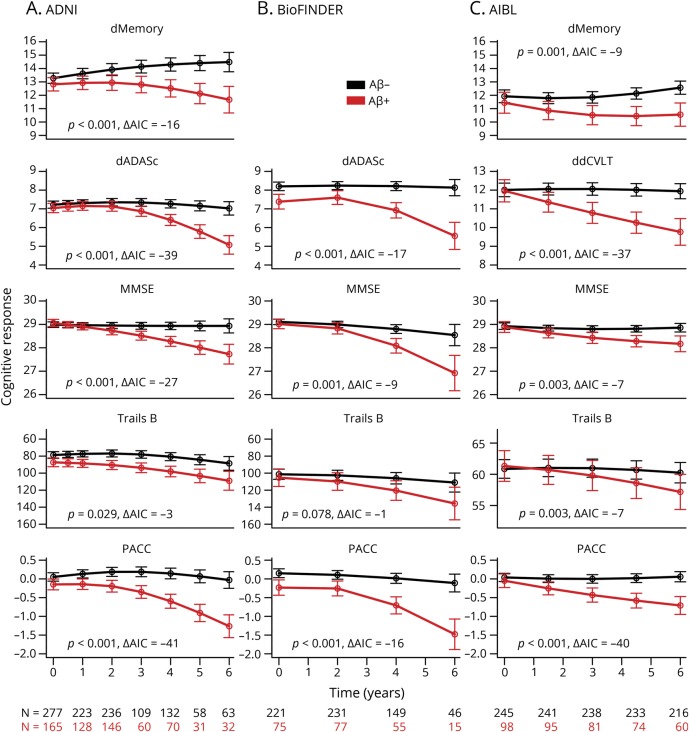
Cognitive change over time Cognitive responses are plotted over time for each β-amyloid (Aβ) group, in each cohort separately: (A) Alzheimer's Disease Neuroimaging Initiative (ADNI), (B) Biomarkers for Identifying Neurodegenerative Disorders Early and Reliably (BioFINDER), and (C) Australian Imaging, Biomarkers & Lifestyle (AIBL). Individual Preclinical Alzheimer's Cognitive Composite (PACC) components are shown as well as the PACC in the bottom row. Akaike information criterion and *p* values are shown in each plot, testing for differences between Aβ groups over time. dMemory = Logical Memory Delayed Recall; MMSE = Mini-Mental State Examination; Trails B = Trail-Making Test B.

Aβ+ participants declined significantly more on the PACC and all individual components of the PACC compared to Aβ− participants, in all 3 cohorts, with the exception of Trails B in BioFINDER (*p* = 0.08). Estimates and longitudinal plots of cognition are shown in figure 1. Estimates of the change from baseline, confidence intervals, and the residual SD for each visit and group are shown in table 2.

**Table 2:**
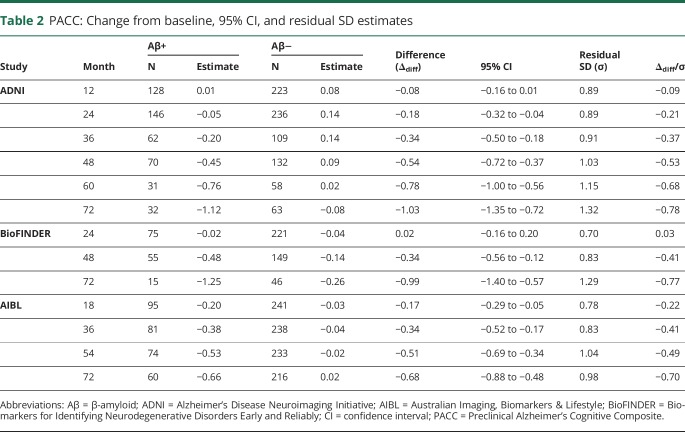
PACC: Change from baseline, 95% CI, and residual SD estimates

At year 4, the Aβ+ groups declined by −0.45 points on the PACC (ADNI), −0.48 points (BioFINDER), and −0.53 points (at 4½ years, AIBL) (table 2). At year 4, the Aβ− group improved 0.09 points on the PACC in ADNI and declined by −0.14 points in BioFINDER and −0.02 points in AIBL.

### Clinical significance

**Figure 2:**
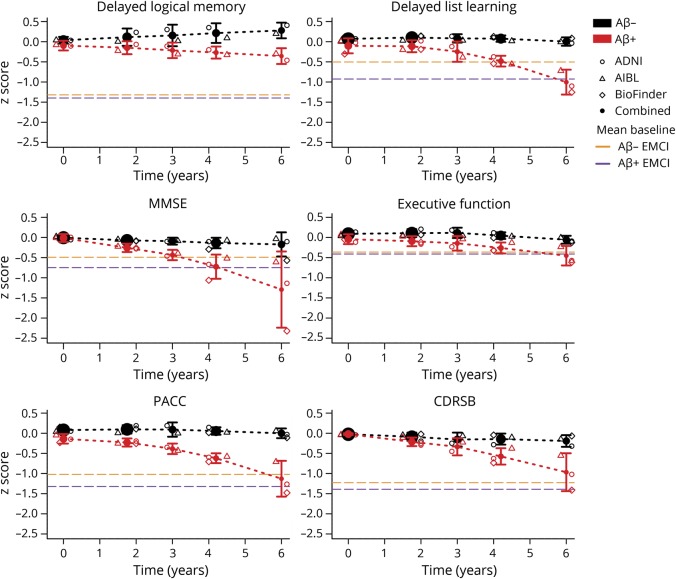
Meta-estimates of change Meta-estimates of change over time are shown by β-amyloid (Aβ) group. Individual cohort estimates are also shown. The mean baseline early mild cognitive impairment scores are shown in dashed purple for Aβ+ and dashed orange for Aβ−. ADNI = Alzheimer's Disease Neuroimaging Initiative; AIBL = Australian Imaging, Biomarkers & Lifestyle; BioFINDER = Biomarkers for Identifying Neurodegenerative Disorders Early and Reliably; CDRSB = CDR sum of boxes; EMCI = early mild cognitive impairment; MMSE = Mini-Mental State Examination; PACC = Preclinical Alzheimer's Cognitive Composite.

To evaluate decline and to characterize what might be considered a clinically significant change, we compared the scores of the cognitively normal participants to the baseline scores of the early MCI participants in ADNI. The mean PACC score in Aβ− and Aβ+ early MCI participants at baseline was −1.01 and −1.30, respectively (figure 2). Six years after baseline, the estimated PACC score combined across cohorts of the preclinical AD groups was midway between the Aβ− and Aβ+ early MCI performance. Similarly, the early MCI Aβ− and Aβ+ scores at baseline on the CDRSB were 1.22 and 1.38, respectively, whereas the preclinical AD groups averaged about 1.0 at 6 years.

On each of the MMSE, delayed list learning, and executive function, the cognitively normal Aβ+ groups averaged worse scores than both MCI groups by 6 years after baseline. The cognitively normal Aβ+ groups did not approach the MCI groups' delayed logical memory scores by 6 years after baseline. Note that delayed logical memory was not available in BioFINDER.

In a sensitivity analysis, 7 early MCI participants who consistently had a global CDR of zero after screening were excluded. The reduced sample scores were slightly worse than the full MCI sample with Aβ− and Aβ+ PACC scores of −1.02 and −1.33, respectively, and CDR-SB scores of 1.23 and 1.39.

### Power

**Figure 3:**
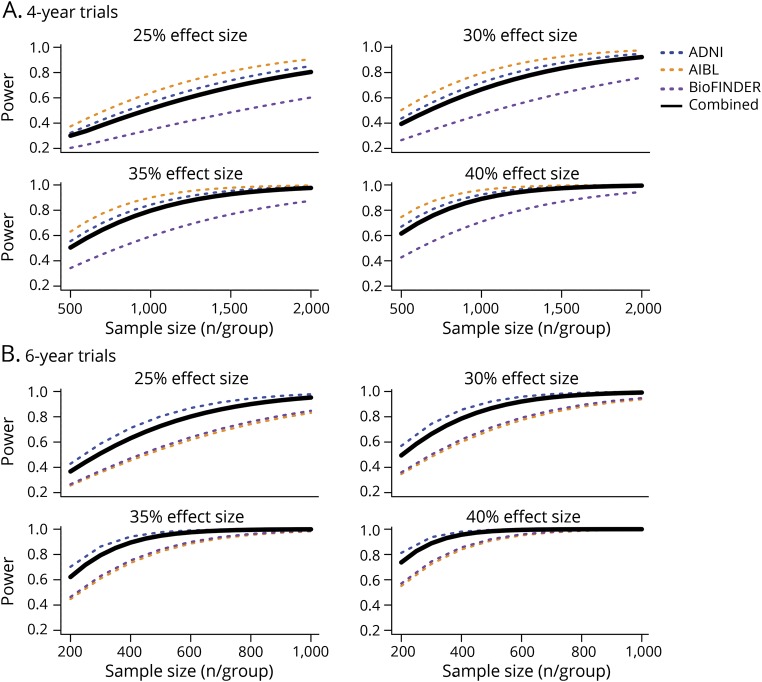
Power Hypothetical clinical trial power is plotted against sample size per treatment group, for each of 4 assumed treatment effect sizes and 2 trial lengths. Individual cohort power curves and a combined estimate are shown. Sample sizes range from (A) 500 to 2,000 per group for 4-year trials and (B) 200 to 1,000 per group for 6-year trials. ADNI = Alzheimer's Disease Neuroimaging Initiative; BioFINDER = Biomarkers for Identifying Neurodegenerative Disorders Early and Reliably.

Using estimates of change and variance, we calculated the power for hypothetical 4- and 6-year clinical trials for each cohort, assuming a 30% dropout rate, and various sample sizes and drug effects (figure 3). In 4-year trials, assuming a 25% drug effect, i.e., a 25% slowing of cognitive decline in the treatment group, the required sample size to reach 80% power was 2,000 per group for the estimate combining all cohorts. Assuming a larger effect size of 35%, the required sample size to reach 80% power was 1,000 per group on average.

In 6-year trials, assuming a 25% drug effect, the required sample size to reach 80% power was about 600 per group for the estimate combining all cohorts. Assuming a 35% effect size, the required sample size to reach 80% power was 300 per group on average.

### Aβ interactions

**Figure 4:**
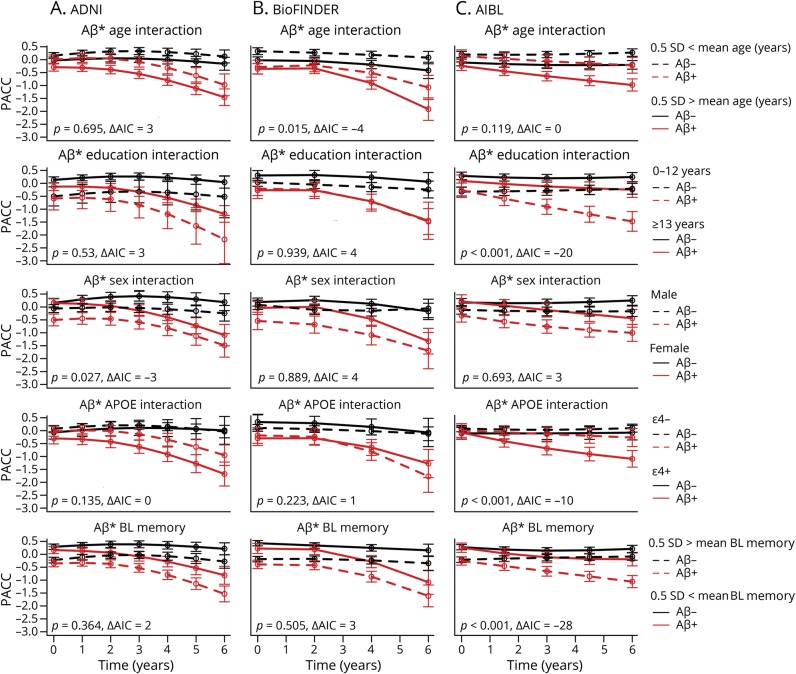
β-amyloid (Aβ) interactions Interactions between Aβ group and age, education, sex, *APOE*, and baseline memory are shown for each cohort: (A) Alzheimer's Disease Neuroimaging Initiative (ADNI), (B) Biomarkers for Identifying Neurodegenerative Disorders Early and Reliably (BioFINDER), (C) Australian Imaging, Biomarkers & Lifestyle (AIBL). Akaike information criterion (AIC) and *p* values are shown in each plot, testing for the significance of interactions in predicting Preclinical Alzheimer's Cognitive Composite (PACC) change over time.

The interactions between Aβ status and baseline factors to predict cognitive decline on the PACC were also assessed. Plots of the amyloid groups at different levels of the significant interacting factors, *p* values, and the change in AIC are shown in figure 4. In AIBL, there were significant interactions between Aβ and education, *APOE* ε4 positivity, and baseline memory. The only significant interaction in ADNI was between Aβ and sex, and the only significant interaction in BioFINDER was between Aβ and age. There were no significant interactions between Aβ and subjective memory complaint (ADNI: *p* = 0.56, AIBL: *p* = 0.87, not available for BioFINDER).

## Discussion

The main findings of this study are (1) cognitively unimpaired Aβ+ participants approach early MCI cognitive performance levels on general cognition and global outcomes, delayed list recall, and executive function by 6 years after baseline; (2) to achieve 80% power in a simulated treatment trial assuming a 25% treatment effect, 2,000 participants/group for a 4-year trial and 600 participants/group for a 6-year trial are required; (3) several baseline factors interacted with Aβ status to predict decline on the PACC including *APOE* ε4 positivity, memory, and education in AIBL; age in BioFINDER; and sex in ADNI, although these findings were all cohort-specific; (4) despite considerable design differences across the cohorts, with large sample sizes and sufficient follow-up time, the cognitively unimpaired Aβ+ groups declined consistently on cognitive composites; (5) Aβ+ groups declined significantly faster on all cognitive tests in all cohorts, with the exception of Trails B in BioFINDER, where the Aβ+ group declined marginally faster (*p* = 0.08), compared to the Aβ− group.

A key question for preclinical AD trials is how to define meaningful outcomes that will support use of therapeutic interventions in people who may remain asymptomatic for many years even without treatment. Traditional AD dementia trials are frequently powered to detect a several-point difference on a global cognitive score (e.g., Alzheimer's Disease Assessment Scale–Cognitive Subscale), as well as a global/functional co-primary outcome to establish clinical meaningfulness.^31^ Post hoc analyses of the first large trials of solanezumab in patients with mild AD showed a 34% reduction of cognitive decline and a 17% reduction of functional decline.^32^ However, these effects were not replicated in a subsequent randomized trial, which failed to show a significant treatment effect, with only an 11% reduction of cognitive decline and 15% reduction of functional decline.^33^ In preclinical AD, the cognitive decline observed over 3–4 years is subtle, and is typically accompanied by little or no functional decline.^34^ However, it has not been clarified what degree of decline would warrant classification as meaningful decline. To benchmark the magnitude of cognitive decline to a measure of clinical meaningfulness, we compared the scores of the cognitively unimpaired participants to those classified as early MCI—a group with incipient functional decline. The separation between these groups was just over 1 SD on the PACC, suggesting that 1 point of additional decline in Aβ+ participants compared to Aβ− participants could be taken as an approximate benchmark for clinically meaningful decline. Combining results across cohorts shows the average Aβ+ participant to have the same PACC score at 6 years post baseline as the average patient with early MCI had at baseline (figure 2). Aβ+ participants also reached MCI level performance at 6 years on the other cognitive outcomes, with the exception of delayed logical memory. Possible explanations for this exception include that this measure was used as inclusion criterion for enrollment. This measure was also not available in BioFINDER, the cohort demonstrating the poorest scores on all measures by the end of follow-up. Finally, delayed logical memory demonstrated a clear practice effect in the Aβ− group (figure 2), with the cognitively unimpaired participants taking this test 6 times over follow-up, compared to one time for the MCI participants.

Based on the PACC estimates, a treatment effect of 40%–50% would be required to delay the cognitive decline of a group of Aβ+ participants from reaching the 1 SD milestone by 3 years. Delaying the cognitive decline equivalent to the level of the average early MCI patient by 3 years may be a clinically meaningful treatment effect. But 40%–50% is a large treatment effect and highlights the difficulties in preclinical AD trial design. However, the observation that clinically meaningful decline is reached within 6 years offers strong support for the use of a cognitive composite in trials that are shorter than 6 years, since short term cognitive decline can be conceptualized as a proxy for downstream functional changes. With meaningful continuous cognitive changes occurring prior to an MCI diagnosis, these results, as well as recent reports,^35^ argue against the use of a time-to-MCI endpoint in preclinical AD trials.

The estimated sample size or trial length requirements are sobering. Previously reported sample size and drug effect requirements of 500/group with a 30%–50% effect size in a 3-year trial were optimistic and based on approximately 20% of the data available in this study.^10^ In order to reliably achieve 80% power for a modest, real-world effect size of 20%–30%, investors in AD research for therapeutics development will have to prepare to support larger and longer trials than are currently envisaged.

There were several significant interactions between Aβ status and baseline factors. However, no interaction was observed in more than one cohort. In AIBL, the combination of Aβ status and low education, *APOE* ε4 positivity, or low baseline memory all led to increased rates of decline on the PACC. Decline in the Aβ+ groups did not depend on *APOE* ε4 status in ADNI or BioFINDER; however, in AIBL, little decline was observed in Aβ+ participants who were not also *APOE* ε4+ (figure 4), as was reported previously.^36,37^ Evidence for additional risk of cognitive decline for individuals who are both Aβ+ and *APOE* ε4+ had been incorporated into the design of a phase 2b/3 trial in preclinical AD (clinicaltrials.gov/ct2/show/NCT02569398); however, this pattern was observed in only one of the 3 cohorts studied here. The additional decline observed in the Aβ+ participants who also had low baseline memory in AIBL is consistent with previous reports.^38^ Still, despite wide separation at baseline, high and low baseline memory (and also high and low education) groups declined in parallel over time in both ADNI and BioFINDER. The lack of replicability of these interactions across cohorts suggests that if there are true underlying effects of these baseline factors that modify the Aβ/cognition relationship, they are mild, or they depend on other/complex interactions. Another possibility is that their identification was the consequence of type I error, although the strength of the associations in AIBL (but not ADNI, reported previously^39^ or BioFINDER) would survive a Bonferroni correction. Our findings caution against relying on interactions between Aβ and demographic/clinical factors when selecting participants for preclinical AD trials.

There were considerable design differences among the 3 study cohorts including differences in geographic region, cognitive measures, visit frequency, and sampling characteristics. Despite these differences, the estimates of decline observed on the PACC in the Aβ+ groups at 4 years were remarkably similar: −0.45 points in ADNI, −0.48 in BioFINDER, and −0.53 (at 4½ years) in AIBL (table 2). Where the cohorts differed was in the change in the Aβ− group: 0.09 in ADNI, −0.14 in BioFINDER, and −0.02 in AIBL. The lower power estimate for BioFINDER for a clinical trial can be traced back to the additional decline observed in the Aβ− group, which may be due in part to including participants with presence of cerebrovascular pathology such as white matter lesions (not excluded from BioFINDER, but may have been excluded from ADNI).^40,41^ Cognitive reserve may also play a role, given the lower levels of education in BioFINDER compared to both ADNI and AIBL.

The Aβ group trajectories on the PACC were similar, though there was variation in the shape of the trajectories for some of the individual components. One design feature that may influence trajectory differences is test frequency. ADNI participants were tested every 6 months over the first year and every year thereafter, whereas AIBL participants were tested every 18 months and BioFINDER, every 24 months. The increased test frequency and higher levels of education in ADNI may have contributed to a tendency to improve over time as seen in dMemory (figure 1). Despite this variation in dMemory slope, Aβ group separation over time was preserved in ADNI and AIBL. For delayed list learning, all Aβ− groups remained stable, and all Aβ+ groups showed similar decline over the total follow-up time. Combining individual components into the composite seemed to mitigate individual domain trajectory differences (figure 2). Overall, the Aβ groups across all 3 cohorts started to diverge reliably around 3 years after baseline.

One of the main limitations of this study is the variation of available measures used to construct the composite cognitive scores (i.e., the PACC) in each of the cohorts. While we included the domains represented in the original PACC, it remains unclear how these substitutions may affect the estimates of Aβ-related cognitive decline. Another limitation is that with strict exclusionary criteria, the participants in these studies have few comorbidities, lack diversity, and do not mirror the general population. Clinical trials frequently use similar exclusionary criteria and may also lack generalizability. An additional limitation to all studies trying to inform disease-modifying AD trials is that without any information regarding potential effects of treatments, the power to detect a hypothetical effect is speculative.

Average cognitively normal Aβ+ participants approach early MCI cognitive performance levels 6 years after baseline. Comparing these 3 cohorts side by side demonstrates that large sample sizes and sufficiently long follow-up times result in consistent estimates of decline in preclinical AD. Despite substantial design and sampling differences, these results support the potential for internationally conducted clinical trials in preclinical AD. However, it is likely that designers of preclinical AD treatment trials will have to prepare for larger and longer trials than are currently considered.

## Glossary

Aβ: β-amyloid
AD: Alzheimer disease
ADNI: Alzheimer's Disease Neuroimaging Initiative
AIBL: Australian Imaging, Biomarkers & Lifestyle
AIC: Akaike information criterion
BioFINDER: Biomarkers for Identifying Neurodegenerative Disorders Early and Reliably
CDR: Clinical Dementia Rating
CDR-SB: CDR sum of boxes
dADASc: Delayed Word Recall from the Alzheimer's Disease Assessment Scale–Cognitive Subscale
dMemory: Logical Memory Delayed Recall
MCI: mild cognitive impairment
MMSE: Mini-Mental State Examination
OR: odds ratio
PACC: Preclinical Alzheimer's Cognitive Composite
PiB: Pittsburgh compound B
SUVR: standardized uptake value ratio
Trails B: Trail-Making Test B
